# Micro-computed tomography analysis of void volume in sealer layer placed using Vibrospiral novel instrument: an *in vitro* study

**DOI:** 10.3389/fdmed.2025.1695090

**Published:** 2025-10-13

**Authors:** Avishikta Banerjee, Mithra Nidarsh Hegde, Kalyanpur Rajat Shenoy

**Affiliations:** ^1^ Department of Conservative Dentistry and Endodontics, AB Shetty Memorial Institute of Dental Sciences, Nitte University, Mangalore, India; ^2^ Independent Researcher, London, United Kingdom

**Keywords:** Vibrospiral, voids, good health and well-being, quality education, industry, innovation and infrastructure

## Abstract

**Aim:**

Endodontic sealers form an intermediary layer between the solid canal filling material, like gutta-percha, and the dentinal wall to fill the voids and porosities between them and establish a fluid-impervious seal, which is essential to prevent reinfection of the canal system. Sealer placement instruments are used to carry and coat the sealer on the walls of the fine, tortuous root canal system. The Vibrospiral is a piezoelectrically activated novel sealer coating instrument that has been designed to place a thin, uniform, void-free sealer layer by its vibratory motion. This study compared the void volume percentage in the sealer layer when placed with the Vibrospiral and two other conventional coating instruments.

**Methodology:**

A total of 36 caries-free natural mandibular premolars were disinfected, decoronated, and biomechanically prepared. They were randomly assigned to one of the three groups (*n* = 12) based on the mode of sealer application: group 1=K-file, group 2=Lentulo spiral, and group 3=Vibrospiral. After sealer placement, the canals were obturated with gutta-percha and the sealer was allowed to set. Samples were then subjected to micro-computed tomography (micro-CT) to assess the void volume. The data acquired were statistically analysed using ANOVA and the Tukey test, and the significance level was set at <0.05.

**Results:**

The findings revealed that the Vibrospiral placed sealer layers with the least void volume percentage overall (3.5%), followed by Lentulo spiral (19.21%) and K-file (22.01%).

**Conclusions:**

The piezoelectrically activated novel sealer placing instrument, the Vibrospiral, has promising potential in effectively placing a relatively non-voided sealer layer.

## Introduction

The creation of a fluid-impermeable seal is the primary goal of root canal obturation. Gutta-percha (GP) is not adherent to the dentin. Endodontic sealers serve as an intermediary layer between the core filling material and the root canal wall to ensure adaptation between them and to fill any remaining voids (1, 2). The sealer is carried into the thin, tortuous root canals with the help of sealer placement instruments, such as Lentulo spiral, master cone, or K-files (3). The quality of the sealer layer placed depends upon the amount of sealer carried into the root canal and the mechanism of action of the sealer placing instrument (3). An ideal sealer layer should be thin, uniform, and devoid of voids (4). The sealer should coat all the walls of the root canal and seal all the lateral canals to ensure a hermetic seal. None of the sealer placing instruments fulfils all the criteria (4, 5).

The Vibrospiral is a novel sealer placement instrument. The device features a spiral working part and a curved shank, allowing attachment to a piezoelectric handpiece. The Vibrospiral is activated by piezoelectric energy, which enables agitation of the sealer, ensuring the placement of a uniform, void-free sealer layer on the canal walls.

This study aimed to assess the effectiveness of the piezoelectrically activated Vibrospiral in placing a root canal sealer layer by comparing it with other sealer placing instruments, such as the K-file (a hand instrument) and a Lentulo spiral (an engine-driven instrument). Micro-computed tomography (micro-CT) was used to quantify the void volume in the sealer layer when sealer was coated on the canal walls using the different instruments.

The null hypothesis states that the Vibrospiral is ineffective in coating root canal walls with minimal voids in the sealer layer.

## Materials and methods

**Figure 1:**
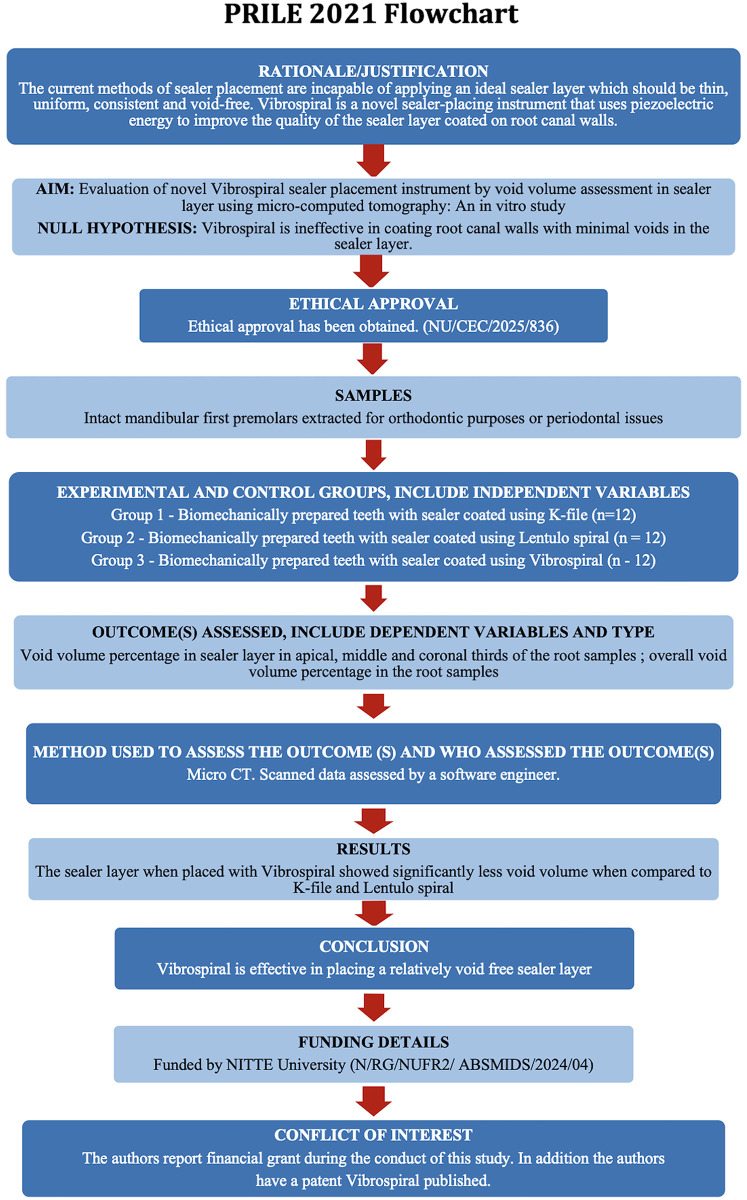
Workflow of the study based on PRILE 2021 guidelines. A schematic representation outlining the sequential steps followed during the study, including sample selection, sample preparation, micro-CT scanning, void segmentation, and statistical analysis, adhering to the PRILE 2021 protocol for standardisation and reproducibility.

The manuscript of this laboratory study has been written according to Preferred Reporting Items for Laboratory studies in Endodontology (PRILE) 2021 guidelines (Figure 1).

This study has received ethical clearance (NU/CEC/2025/836).

### Sample size calculation

Based on an article by Huang et al. (6), using a pooled standard deviation of void volume in AH Plus of 0.4116 mm^3^ across the apical, middle, and coronal thirds, a mean difference of 0.55, an effect size of 1.34, an alpha error of 5%, and a power of 80% for a two-sided test, the required sample size was calculated as 12 per group (three groups, *n*=36). nMaster software (Version 2) was used for the calculation.

### Sample selection and preparation

A total of 36 caries-free human mandibular premolars extracted for orthodontic reasons were collected. They were screened for defects, cleaned, and disinfected. Teeth were handled as per the recommendations laid down by the Occupational Safety and Health Administration (OSHA) and the Centre for Disease Control and Prevention (CDC) (7, 8). While handling the extracted teeth, personal protective equipment (PPE) was worn. Using an ultrasonic cleaner, the teeth were cleared of tissue, debris, blood, and calculus before being placed in a sturdy container with formalin and distilled water. The biohazard emblem was attached to each container and the lids were securely fastened. Before use, the teeth were heat-sterilised for 40 min in an autoclave cycle. Digital radiographs were taken from the buccolingual and mesiodistal aspects to ensure anatomic comparability between the teeth. Only teeth with a single root and a straight single canal were included in the study. Teeth with caries, fracture, root resorption, or developmental anomalies were excluded.

All sample preparations were carried out by a single operator to ensure procedural consistency.

A diamond disc was used to decoronate all teeth and standardise the root length at 12 mm. An ISO no. 10 K-file (Dentsply Maillefer) was inserted till just visible beyond the apex to establish a glide path and 0.5 mm was subtracted from this length to establish the working length. The canals were biomechanically prepared using the crown-down technique with the ProTaper (Dentsply Sirona) rotary file system till file size F3. Canals were irrigated with 5 mL of 3% NaClO, alternating with 1 mL of 17% EDTA during the biomechanical preparation. Final irrigation was carried out with sterile water. The canals were dried with paper points (Dentsply Maillefer).

### Sealer manipulation

The biomechanically prepared roots were randomly divided into three groups, with 12 samples in each group, based on the mode of sealer placement. The manufacturer's instructions were followed to mix AH Plus sealer with a spatula and a mixing pad.

### Sealer quantification

To ensure consistent sealer application across all samples, two methods—direct and indirect—were employed to quantify the sealer.

In the direct method, performed before obturation, the sealer was first manipulated and weighed using an analytical balance. An equal amount of sealer (by weight) was then applied to the canal walls of each root specimen, ensuring uniform distribution.

An indirect method was subsequently used to verify the amount of sealer placed after obturation. For each sample, the weight of the biomechanically prepared tooth and the gutta-percha (GP) cones designated for obturation were recorded. After obturation, the weight of the sheared-off GP and the obturated tooth sample was measured. Using these recorded values, the net weight of the sealer in each sample was calculated by subtracting the combined weight of the GP and the prepared tooth from the total post-obturation weight. This served as a measure to ensure that a similar amount of sealer was present in all specimens.

### Sealer placement

In group 1, sealer was coated on the root canal walls using a K-file, in group 2 with a Lentulo spiral, and in group 3 with a Vibrospiral.

In group 1, the stopper on the K-file was adjusted to the working length. Sealer was loaded on the K-file and introduced into the root canal. The sealer was then coated on the root canal walls by manually rotating the file in an anticlockwise direction, while moving the instrument along the walls for 5 s, followed by withdrawal of the instrument from the canal.

In group 2, a #3 Lentulo spiral was used to carry and coat the sealer into the canal. Sealer was loaded on the Lentulo spiral and it was introduced into the canal. The instrument was then activated to a speed of 300 rpm and was used in gentle up-and-down strokes for 5 s, till the working length.

In group 3, the sealer was coated on the canal walls using a Vibrospiral. It was connected to an ultrasonic unit set at a frequency of 30 kHz. After loading the sealer on the instrument, it was inserted into the root canal till the working length. It was then activated for 5 s and gently moved around the canal in an up-and-down motion to coat the sealer on the canal walls.

After the coating of sealer on the canal walls, F3 size Gutta-percha points (Dentsply Maillefer) were used to obturate the canals using the single-cone obturation technique. For temporary restoration of the coronal access preparation, Cavit was used. The samples were then stored in 100% humidity at 37°C for 7 days, allowing the sealer to set.

### Micro-CT acquisition

Micro-CT scanning was performed by a single operator blinded to the groups.

**Figure 2:**
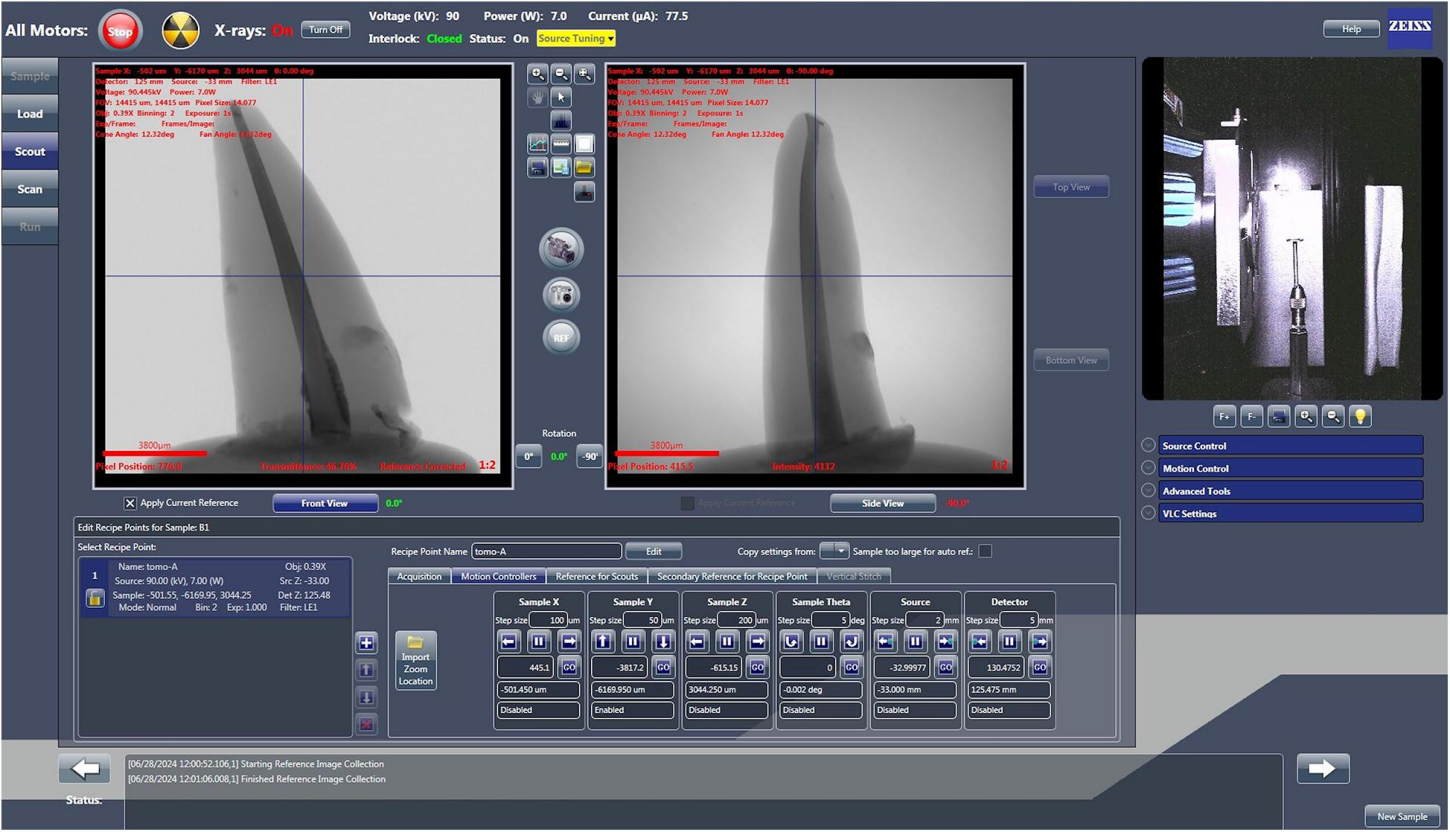
Scout and scan control system interface displaying scanning parameters. Screenshot of the Scout and Scan Control System version 12 (Zeiss, Germany), showing the specific parameters configured for scanning the root canal samples.

The obturated roots were scanned with X-ray Microscopy (XRM) using micro-computed tomography (micro-CT) (Zeiss XRadia Versa 500, Germany) equipped with sub-micro voxel resolution. A voltage of 70 kV, power of 6 W, and current of 85.5 µA with an exposure time of 1 s and an objective of 0.39 X were used for the scan. The LE2 filter, comprising 0.21-mm-thick aluminium, was chosen to eliminate X-rays with low energy. The scans had a pixel size of 13.609. Approximately 1 h was required to scan one root sample. The manufacturer's guidelines and results of preliminary scanning and reconstruction tests served as the basis for setting the beam hardening correction and optimal contrast limits (Figure 2).

### Imaging reconstruction and processing for void volume estimation

Analysis of the micro-CT data was carried out independently by an evaluator under blinded conditions.

**Figure 3:**
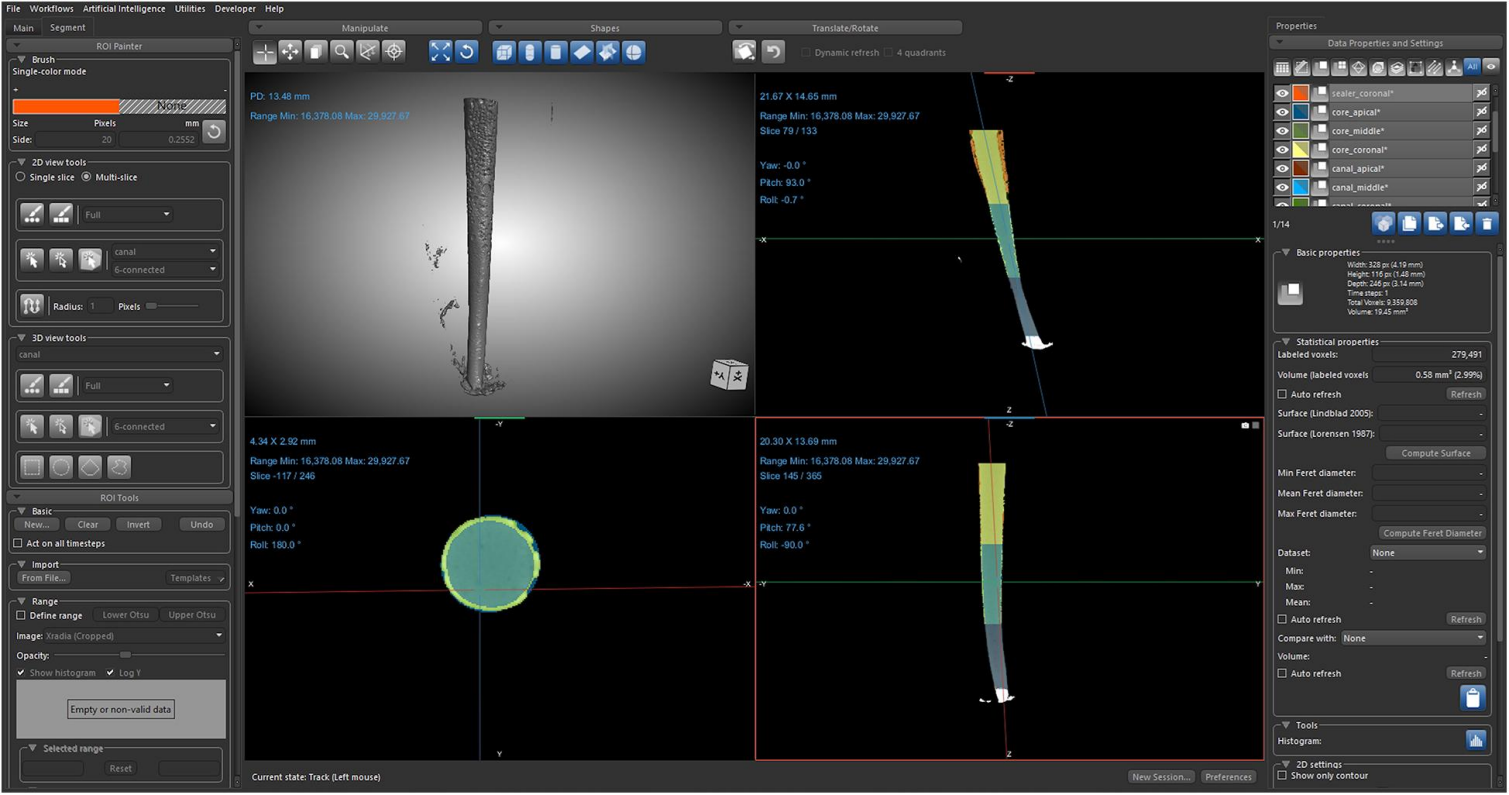
Void volume acquisition using Dragonfly software. Representative image from Dragonfly software depicting the segmentation and quantification of voids in the sealer layer, derived from the scanned dataset.

Data acquisition and reconstruction were carried out using Scout and Scan control system version 12 (Zeiss, Germany). The data were acquired in DICOM format and imported into Dragonfly 3D World version 2024.1 (Comet Tech Canada Inc.) for image processing and volumetric analysis (6). Each dataset comprised approximately 1,015 slices, with a matrix dimension of 996 × 1,024 pixels and a total voxel count of approximately 1.03 billion, corresponding to a reconstructed volume of 2,150.82 mm^3^. To isolate the region of interest (ROI), each dataset was initially cropped to include only the filled root canal space. Image intensities were manually adjusted across all three orthogonal planes (axial, sagittal, and coronal) to enhance the contrast between the obturation materials and surrounding voids (9). The sealer exhibited the highest radiodensity, followed by the core material, while voids and background regions appeared as low-intensity (black) voxels (10), thus enabling clear visual differentiation. Subsequent segmentation was conducted in three stages: canal segmentation was performed using intensity-based thresholding under the ROI tool. A preliminary mask was generated by excluding low-intensity (void and background) voxels. Manual refinement was then performed using the ROI Painter tool to correct under- and over-segmented areas. Sealer segmentation was performed by defining an upper-intensity threshold that encompassed the brightest voxel values (10) corresponding to the radiopaque sealer layer, followed by manual refinement. Core segmentation targeted intermediate-intensity grey regions (10), representing gutta-percha, adjusted similarly through visual thresholding followed by manual refinement. These thresholds were empirically determined by visual observation and remained sample-specific due to inherent variation in radiodensity. No fixed greyscale range was applied across datasets. Each segmented layer's volume (in mm^3^) was automatically computed using Dragonfly's “Basic Properties” panel (Figure 3).

The canal, sealer, and core volume were derived from the micro-CT scans. The void volume (6) within each sample was calculated indirectly by subtracting the combined volumes of the segmented sealer and core materials from the total segmented canal volume:

VoidVolume=CanalVolume−(SealerVolume+CoreVolume)

To ensure the reproducibility and reliability of the segmentation, an independent second observer reviewed each segmented dataset and suggested necessary corrections. Discrepancies were resolved through consensus to minimise observer bias.

The void volume percentage was calculated as the ratio of the void volume to the total canal volume.

### Statistical analysis

The statistical analysis was performed using Statistical Package for Social Science software version 23.0 (SPSS, IBM Corp., Armonk, NY, USA). The data were statistically analysed using ANOVA and the Tukey test. The statistical significance level was set at <0.05.

## Results

**Figure 4:**
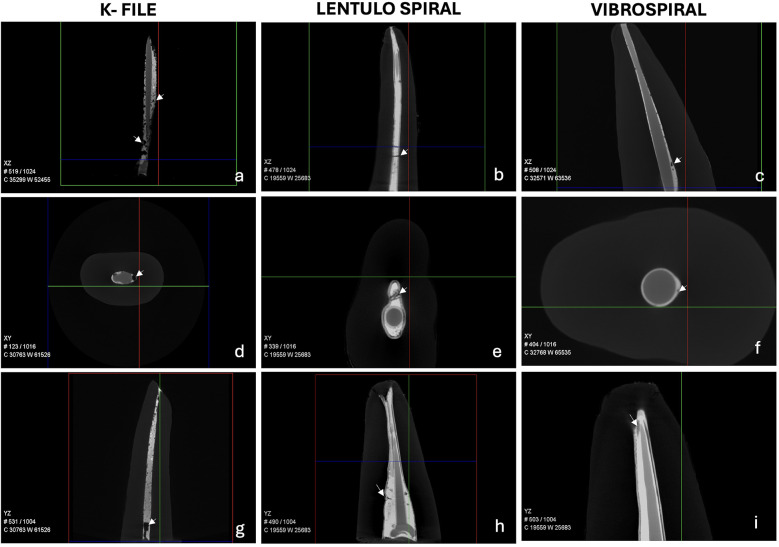
Representative scanned tooth samples showing gutta-percha (GP), sealer, and voids in the sealer layer. Multiplanar reconstructions illustrating the spatial distribution of voids (indicated by arrows) with three different sealer placement techniques. **(a–c)** XZ plane: Sealer placed using the K-file, Lentulo spiral, and Vibrospiral, respectively. **(d–f)** XY plane: Sealer placed using the K-file, Lentulo spiral, and Vibrospiral, respectively. **(g–i)** YZ plane: Sealer placed using the K-file, Lentulo spiral, and Vibrospiral, respectively.

**Figure 5:**
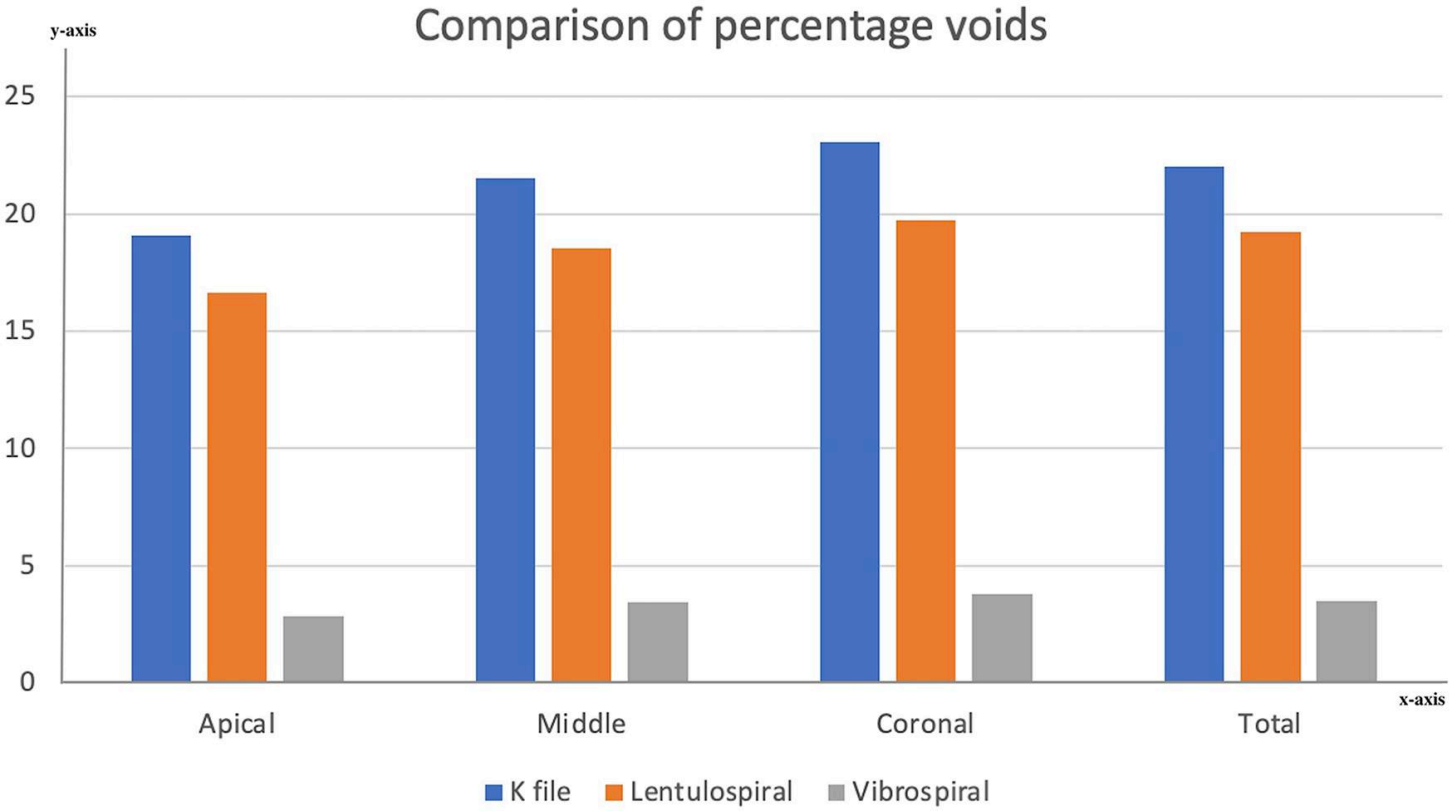
Bar graph comparing void volume percentages across root thirds and the entire root sample among different sealer placement techniques. Visual representation of void distribution in apical, middle, and coronal thirds, as well as total void volume in the root canal system.

Three-dimensional reconstructions of the obturated teeth were obtained using image processing software. Radiolucent voids were observed along the entire length of the radiopaque sealer layer in all samples (Figure 4). The percentage of void volume was measured in the apical, middle, and coronal thirds, as well as the total void volume for each sample, and compared across three sealer placement techniques: K-file, Lentulo spiral, and Vibrospiral (Figure 5).

Mean void volume percentages and standard deviations for each root third and the entire root are presented in Table 1.

**Table 1:** Intergroup comparison of mean void volume percentage (%) in the sealer layer at the apical, middle, and coronal thirds of the root canal, with each group representing a different sealer placement technique.

Group	Apical		Middle		Coronal		Overall	
	Mean	SD	Mean	SD	Mean	SD	Mean	SD
K-File (Group 1)	19.08	1.24	21.50	1.35	23.04	1.64	22.01	1.16
Lentulo spiral (Group 2)	16.65	6.54	18.51	6.50	19.74	1.58	19.21	2.38
Vibrospiral (Group 3)	2.86	0.72	3.41	0.78	3.78	0.54	3.50	0.41

SD, standard deviation.

One-way ANOVA revealed significant differences among the three groups in all sections and overall. *Post-hoc* multiple comparison tests showed that the Vibrospiral produced significantly lower void volumes compared to both the K-file and Lentulo spiral across all regions (*p* < 0.05).

In addition, the K-file and Lentulo spiral differed significantly in void volume percentage in the coronal third and overall. However, no statistically significant difference was observed between the K-file and Lentulo spiral groups in the apical and middle thirds (Table 2).

**Table 2:** Intergroup comparison of statistical significance (*p*-values) in void volume percentage within the sealer layer in apical, middle, and coronal sections.

Group	Apical	Middle	Coronal	Overall
	*P*-value	*P*-value	*P*-value	*P*-value
Group 1 and Group 3	0.000*	0.000*	0.000*	0.000*
Group 2 and Group 3	0.000*	0.000*	0.000*	0.000*
Group 1 and Group 2	0.284 (ns)	0.156 (ns)	0.000*	0.000*

*Statistically significant at *p* ≤ 0.05.

ns, not significant.

## Discussion

A sealer layer sandwiched between solid core filling material (e.g., Gutta-percha) and dentin fills the space between them (11). During the application of sealer on canal walls, voids often get entrapped in the sealer layer, which serves as a niche for bacteria (12). If the seal in the root canal is improper, proliferating microbes residing in voids can migrate to the periapex, leading to persistent symptoms and an unsuccessful endodontic treatment (6). Hence, the sealer layer should ideally be free of voids, ensuring complete filling of the root canal space, leading to a three-dimensional hermetic seal and good long-term prognosis (13). The quality of the sealer layer depends on the instrument used for sealer application and its mode of activation (3). Various instruments have been used to apply sealer on the root canal walls (5, 14). However, none of the existing sealer coating instruments can place a void-free sealer layer (4, 11).

The Vibrospiral, a novel sealer placing instrument, was designed to overcome the drawbacks of the pre-existing sealer coating instruments. This study supports Sustainable Development Goal 3 (Good Health and Well-Being) by addressing the quality of sealer placement in root canal therapy, which directly influences treatment success and long-term patient outcomes.

The Vibrospiral is made of corrosion-resistant 18-8 stainless steel, which is composed of 18% chromium and 8% nickel. It has an L-shaped non-working shank, one end of which is connected to the handpiece of a piezoelectric unit, while the other end forms the working component, comprising a short straight wire continuing into a tapered spring of 20 mm length. The length of the spiral is adequate to ensure that the sealer is carried to the apical third of the root canal. The spiral design renders flexibility to the instrument so that it can be easily inserted into the curvilinear root canals. The taper of the spiral provides additional benefit by conforming to the natural apical taper of root canals. The junction at the shank and the straight wire portion of the working component forms the weakest point of the instrument. This allows easy retrieval of the instrument from the canal in case of a fracture, minimising potential complications.

This study evaluates the effectiveness of the Vibrospiral in coating sealer by comparing it with other instruments such as the K-file and Lentulo spiral. The study is consistent with Sustainable Development Goal 9 (Industry, Innovation, and Infrastructure), as the Vibrospiral is a novel instrument with a unique design, for which a patent has been published.

The filling quality of root canals can be assessed using different techniques, such as dye leakage, assessing penetration of microbes, and evaluating histological sections (15, 16). In the dye penetration technique, dye infiltrates around voids and into the root filling material, making it difficult to quantify void volume. During the preparation of sectioned samples for histological evaluation, it was found that there was significant tissue loss due to the sectioning process, which led to reduced material being left to assess. Microbiological penetration was a more biologically relevant outcome. Moreover, it only revealed voids extending through the entire length of the canal (17). The conventional CT scans render images with low resolution. Micro-computed tomography (micro-CT) enables precise, non-invasive evaluation of root canal morphology. It provides high-resolution data that can be reconstructed in any plane and visualised as two-dimensional slices or three-dimensional renderings. Internal and external anatomies can be evaluated concurrently or independently, supporting qualitative and quantitative analysis. Hence, micro-CT was the tool of choice for this study to render void volume in the sealer layer (18). This study contributes to Sustainable Development Goal 4 (Quality Education), as the application of micro-CT provides a highly accurate analysis of void volume in the sealer layer, thereby enhancing understanding of endodontic procedures for both research and educational purposes.

AH Plus is dimensionally stable, has low solubility, and good flow (19). Its use has been widely recommended based on extensive long-term clinical experience and it is considered the gold standard among sealers (20). Hence, AH Plus sealer was used for the study.

The results of the study revealed that the void volume in the sealer layer was lowest when the Vibrospiral was used to coat the sealer, followed by Lentulo spiral and the K-file.

The void volume results were expressed as the percentage of voids in the root canal space; hence, the variation in the root canal volume among different samples due to varied anatomy was nullified.

The K-file was operated by hand to coat the sealer on the root canal walls. Since there was no mechanised agitation of the sealer, the presence of voids in the sealer layer was inevitable (14). A study conducted by Kahn et al. showed similar results, where it was concluded that the Lentulo spiral, ultrasonic, and sonic files were more effective in coating sealer than the K-file and paper points (14).

Another conventional sealer placing instrument, the Lentulo spiral, when activated, has a rotatory motion that deposits sealer on the canal walls by its centrifugal action. Although this method ensures agitation of the sealer, the high-speed rotary motion entraps air in the sealer, which results in a void-filled, uneven sealer layer (5, 21).

The Vibrospiral is a novel endodontic instrument designed to coat a relatively voidless sealer layer on the canal walls. It features a tapered, spring-like working tip extending from a curved, non-working shank and is activated using a piezoelectric handpiece. Initially operated at a high frequency of 32–36 kHz, the device generated excessive heat, leading to irreversible alteration of the sealer’s properties and fracture of the spiral tip. To address this, the activation frequency was reduced to 30 kHz, which proved effective. The tapered configuration of the Vibrospiral conforms to the prepared root canal morphology, while its spiral design effectively carries the sealer into the canal and ensures uniform coating of the canal walls. The flexible, spring-like structure allows it to navigate narrow and curved canals and ensures uniform coating of the canal walls. In contrast, traditional ultrasonic instruments with rigid, straight designs lack flexibility and the capacity to transport sealers into complex canal systems effectively. As a result, the Vibrospiral offers a significant design advantage over conventional ultrasonic applicators.

Piezoelectric activation of the Vibrospiral is characterised by high frequency and low oscillation amplitude. Ultrasonic energy creates several nodes and internodes along the length of the Vibrospiral, generating strong currents (22) and delivering energy to the sealer (23). The heat produced in the process is sufficient to increase sealer fluidity (24, 25). Ultrasonic activation amplifies the adaptation of the sealer by increasing its pressure against the canal walls, thereby reducing the incidence of voids and improving the sealing of anatomical complexities and irregularities (26, 27).

Similar results were seen in other studies where ultrasonic stimulation was found to improve the quality of the sealer layer placed (5, 26, 28–32). Hoen et al. compared sealer placement with ultrasonic and hand instruments, concluding that canal wall coverage with sealers was superior with ultrasonic instruments compared to hand instruments (33).

The null hypothesis was rejected as the Vibrospiral proved to be an effective instrument in coating root canal walls with sealer.

The Vibrospiral helps in achieving a comprehensive three-dimensional seal in endodontic procedures, thereby promoting good health and well-being in patients. In addition, this study supports quality education by expanding dental professionals' understanding of advanced tools and methods that enhance the effectiveness of endodontic care.

### Clinical significance

The Vibrospiral places a sealer layer with minimal voids, which increases the success of root canal treatment by reducing the chances of reinfection from bacteria that may persist in residual voids.

### Limitations

The present study was conducted under *in vitro* conditions, which do not fully replicate the complex biological and mechanical environment of the oral cavity, such as masticatory load, temperature cycling, and saliva/biofilm effects. The performance of the Vibrospiral in curved or multi-rooted teeth was not assessed, limiting the generalisability of the findings to more complex clinical scenarios (34). Further *in vivo* studies are needed to validate the clinical relevance of the findings.

### Future scope

Future studies should assess additional parameters, including sealer penetration depth into dentinal tubules and sealer layer thickness, to enable a more comprehensive evaluation of performance of the Vibrospiral. Finite element analysis (FEA) of the Vibrospiral may help optimise its design and quantify heat generation, which can then be examined for potential effects on sealer chemistry.

## Conclusion

The Vibrospiral places a sealer layer with fewer voids compared to traditional instruments such as the K-file and Lentulo spiral, owing to its innovative design and piezoelectric activation. This improved sealer placement may contribute significantly to the overall success of root canal treatment.

## Data Availability

The raw data supporting the conclusions of this article will be made available by the authors, without undue reservation.
